# CSM‐Potential2: A comprehensive deep learning platform for the analysis of protein interacting interfaces

**DOI:** 10.1002/prot.26615

**Published:** 2023-10-23

**Authors:** Carlos H. M. Rodrigues, David B. Ascher

**Affiliations:** ^1^ Computational Biology and Clinical Informatics, Baker Heart and Diabetes Institute Melbourne Victoria Australia; ^2^ School of Chemistry and Molecular Biosciences University of Queensland Brisbane Queensland Australia

**Keywords:** deep learning, protein structure, protein–ligand, protein‐NA, protein–protein

## Abstract

Proteins are molecular machinery that participate in virtually all essential biological functions within the cell, which are tightly related to their 3D structure. The importance of understanding protein structure–function relationship is highlighted by the exponential growth of experimental structures, which has been greatly expanded by recent breakthroughs in protein structure prediction, most notably RosettaFold, and AlphaFold2. These advances have prompted the development of several computational approaches that leverage these data sources to explore potential biological interactions. However, most methods are generally limited to analysis of single types of interactions, such as protein–protein or protein–ligand interactions, and their complexity limits the usability to expert users. Here we report CSM‐Potential2, a deep learning platform for the analysis of binding interfaces on protein structures. In addition to prediction of protein–protein interactions binding sites and classification of biological ligands, our new platform incorporates prediction of interactions with nucleic acids at the residue level and allows for ligand transplantation based on sequence and structure similarity to experimentally determined structures. We anticipate our platform to be a valuable resource that provides easy access to a range of state‐of‐the‐art methods to expert and non‐expert users for the study of biological interactions. Our tool is freely available as an easy‐to‐use web server and API available at https://biosig.lab.uq.edu.au/csm_potential.

## 1 INTRODUCTION

Proteins mediate a range of critical biological functions in the cell, such as the transport of molecules in and out,^1^ identification of pathogens and recruitment of T‐cells to kill them,^2^ and cell growth,^3^, ^4^ via tightly coordinated interactions with other molecules. Understanding of the molecular mechanisms of how the different molecules interact represents an attractive way of potentially utilize these interacting interface regions for the development of new drugs. In addition, increasing our understanding of how specific pathways of interactions occur may help elucidate how diseases affect these interactions and how to better treat them.

The importance of studying these interacting interfaces is also evidenced by the great diversity and variety of computational methods proposed in recent years, leveraging large amounts of protein sequence and structure available in databases such as Uniprot^5^ and the Protein Data Bank (PDB),^6^ and more recently the AlphaFold database^7^ and the ESM Metagenomic Atlas.^8^ These have ranged from tools utilizing either only protein sequence^9^, ^10^ to more sophisticated approaches that tried to combine sequence and structural information to improve their performance.^11^ However, despite the number of tools available for studying the interaction of proteins with other molecules, most methods explore specific interaction types (only protein–protein, protein‐NA, or protein ligand interactions) and lack user‐friendly interfaces for nonexpert users. To leverage the potential of these approaches with some of the techniques developed *in‐house* by our research group, we previously released CSM‐Potential,^12^ which consolidates our graph‐based approach with geometric deep learning to identify regions of a protein surface that are likely to mediate protein–protein and protein–ligand interactions in order to provide a link between 3D structure and biological function.

**FIGURE 1:**
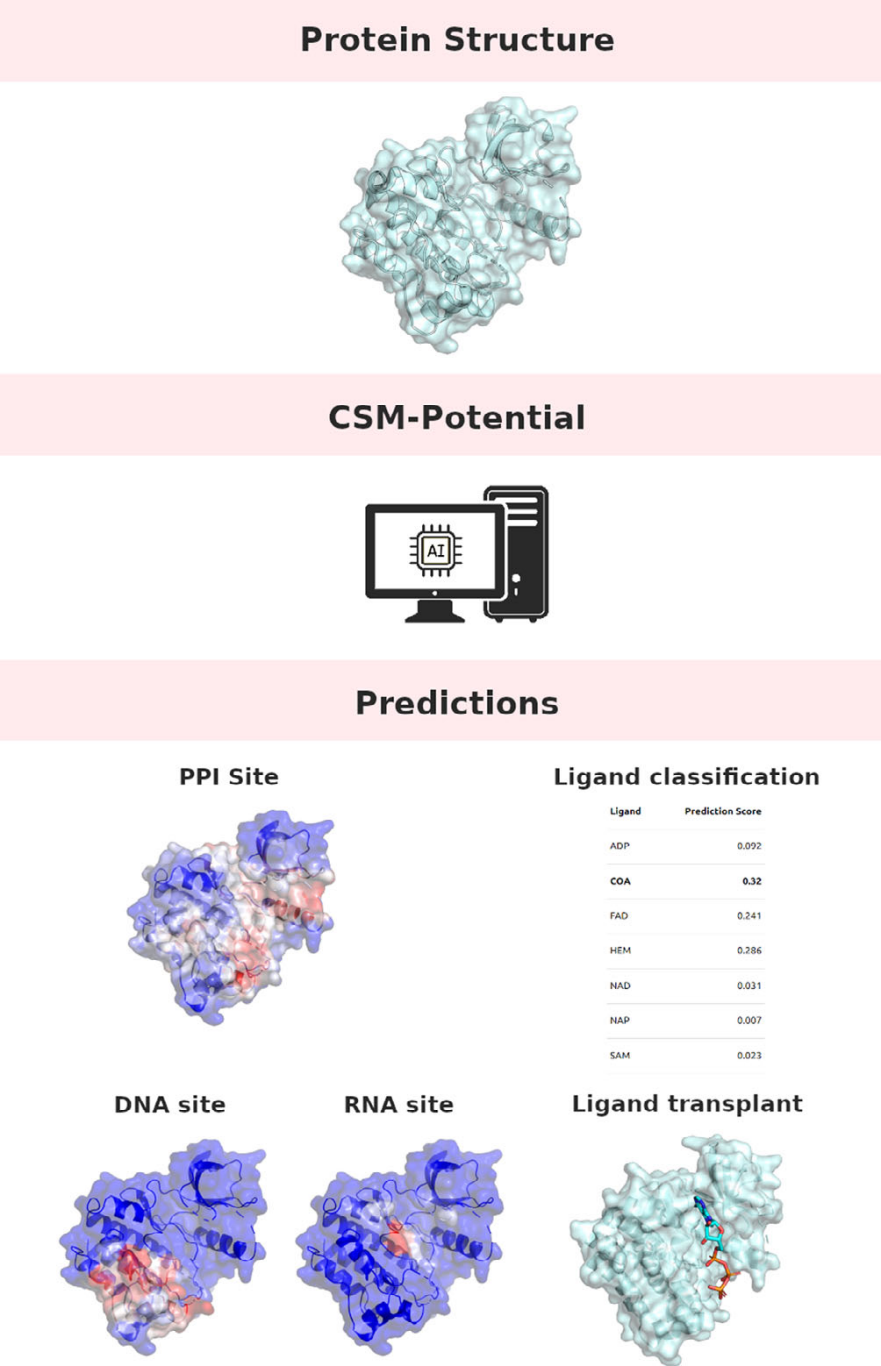
CSM‐potential a deep‐learning platform for the study of protein interactions. The CSM‐Potential platform offers easy access to a range of state‐of‐the‐art approaches to help study the interactions of proteins with a diversity of other molecules, including other proteins, nucleic acids, and ligands.

Here we propose CSM‐Potential2 (Figure 1), an update to our previous implementation to consolidate our tool as a deep learning platform for the study of protein binding interfaces. In addition to prediction of PPI binding interfaces at a residue level and classification of biological ligands for specific pocket regions, here we incorporated prediction of nucleic acids (DNA and RNA) binding interfaces and ligand transplantation based on structure and sequence similarity.

## 2 RESULTS

**FIGURE 2:**
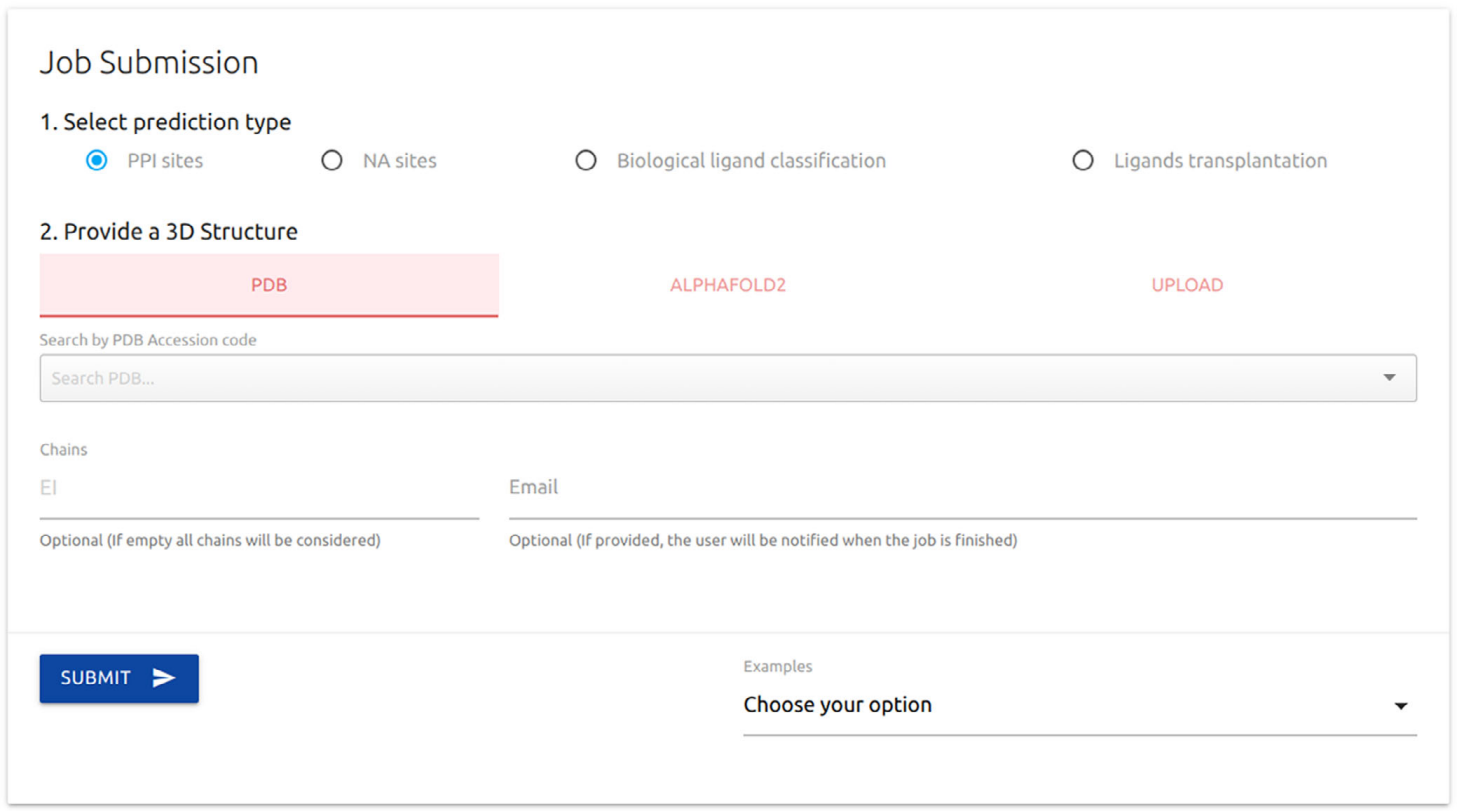
Input page for CSM‐Potential2. Our deep‐learning platform offers fur different types of analysis: Identification of (1) PPI and (2) NA binding sites (3); biological ligand classification, where users can select specific pocket regions on a given protein structure and access which of seven ligands (ADP, COA, FAD, HEM, NAD, NAP, and SAM) is more likely to binding; and (4) ligands transplantation using the AlphaFill algorithm. For all options, CSM‐Potential2 requires users to provide a protein structure via searching structures available on the PDB and AlphaFold databases or uploading a structure in PDB format. For multimeric structures, users may choose only specific monomers using the “Chains” field. If provided, an email will be sent to notify users once the results are ready. Examples for each submission type are also available.

Our CSM‐Potential platform is freely available as a web server and API at https://biosig.lab.uq.edu.au/csm_potential. All four types of predictions require the user to provide a protein structure via querying for available entries in the PDB and AlphaFold^7^ databases or uploading a file in PDB format (Figure 2). If the structure is multimeric, users have the option to select specific monomers via the “Chains” input. If no option is provided all chains will be considered for the analysis, except for prediction of NA binding sites, which will select the first chain in order of appearance in the input structure. Furthermore, if provided an email will be sent after the job finishes processing to notify users their results are ready.

**FIGURE 3:**
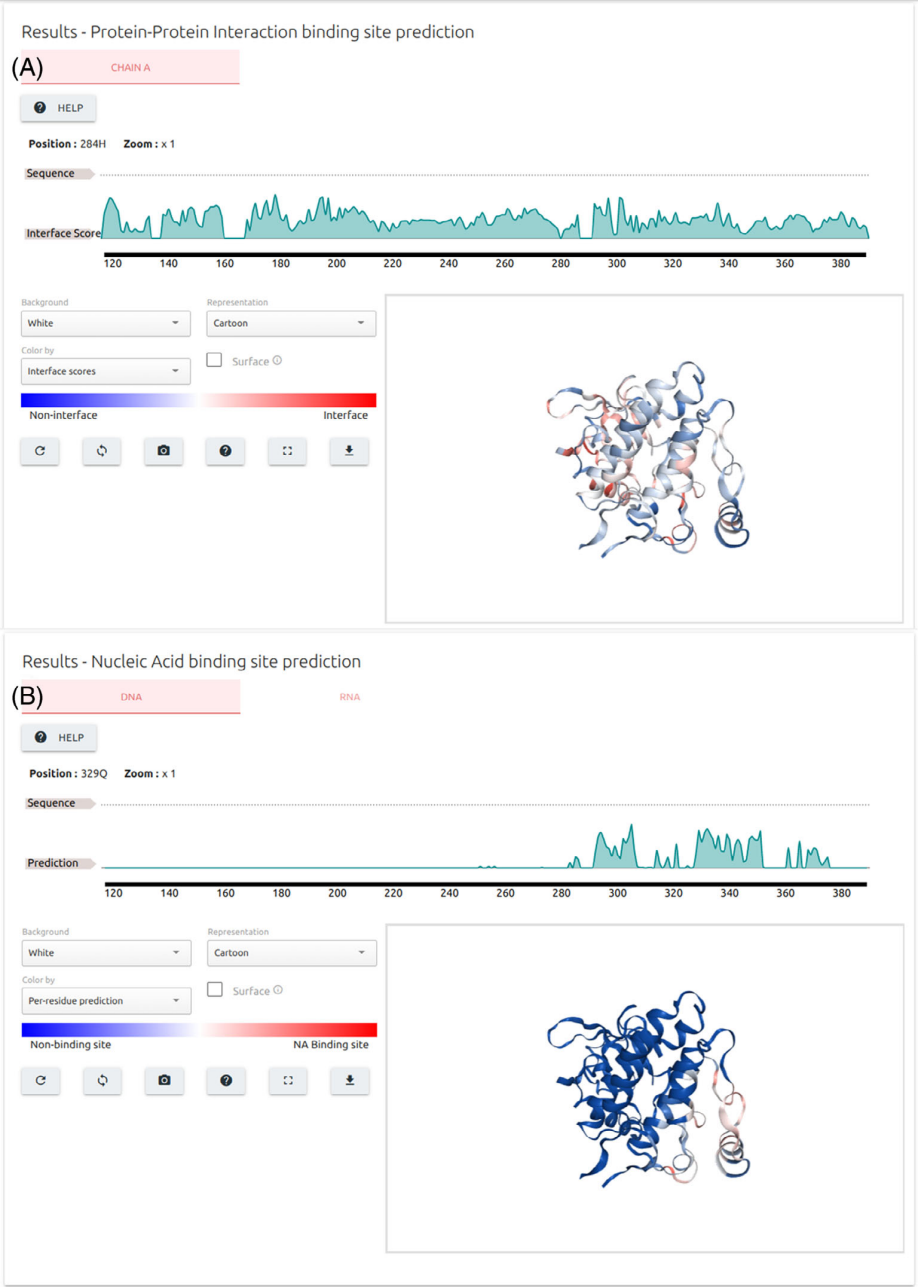
CSM‐Potential2 output page for identification of binding sites. Results for PPI (A) and nucleic acids (B) binding site identification are summarized at residue level using a sequence plot on the top of the page and mapped onto the input protein structure colored from blue (low probability) to red (high probability). For multimeric structures, the sequence plots for PPI binding sites predictions for each monomer are separated into tabs. Similarly, results for DNA and RNA binding site predictions are also separated into tables A set of controls is available for customization of the 3D interactive viewer including downloading the input structure with predictions annotated in the b‐factor column.

Results for identification of PPI and NA binding sites (Figure 3) are summarized at residue level into a sequence plot at the top of the output page and mapped to the input 3D structure using the NGLViewer JavaScript library.^13^ In case the input protein structure is multimeric, sequence plots are generated for each monomer and separated by tabs. Results for NA binding site prediction are also separated into tabs for DNA and RNA. In both cases, a series of controls are provided to help users to customize the interactive 3D viewer to their liking and download input structure with predictions mapped to the b‐factor column for further analysis.

**FIGURE 4:**
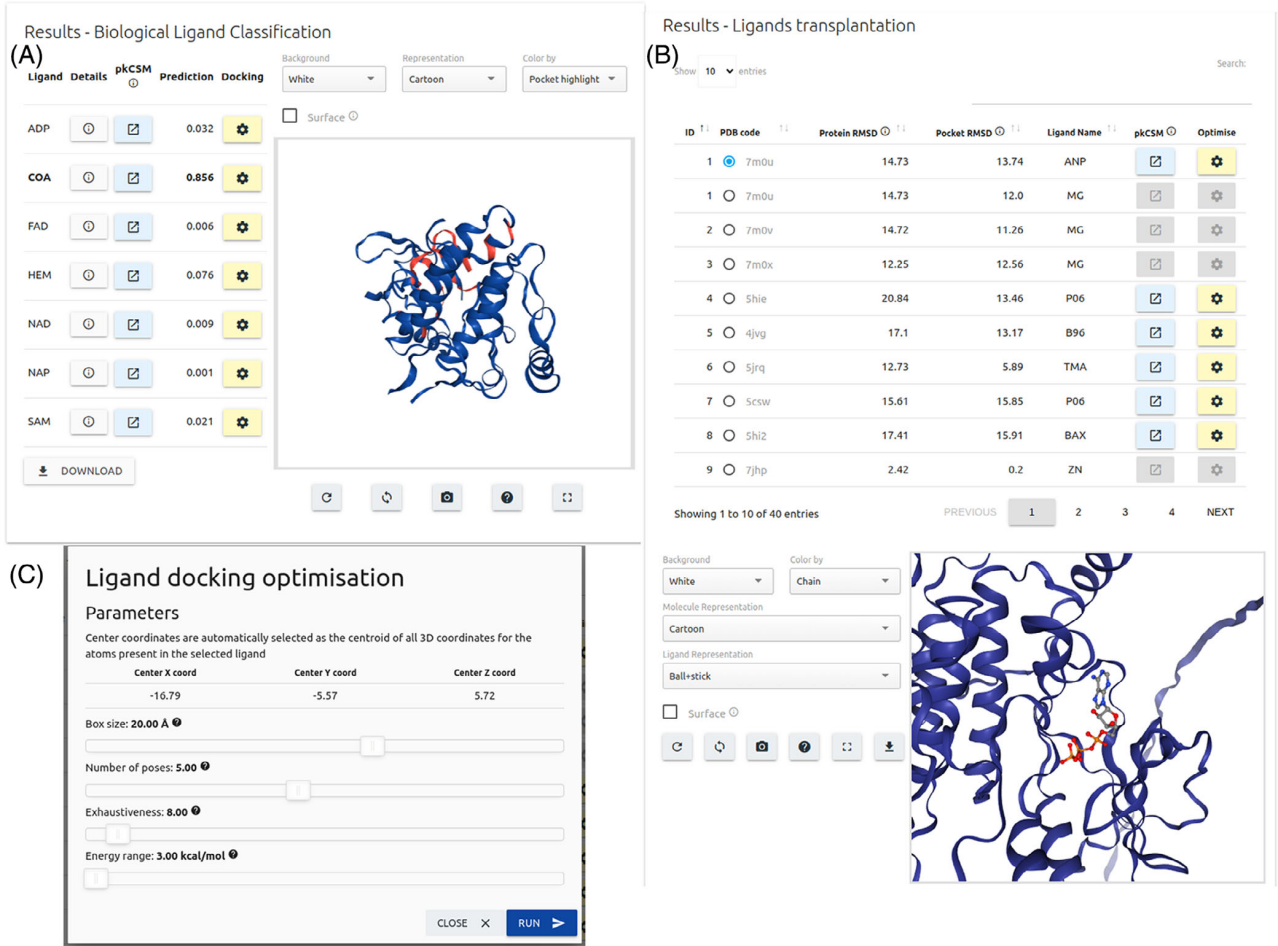
CSM‐Potential2 output page for biological ligand classification and ligand transplantation. Results for the biological ligand classification option (A) are shown as predicted probability scores for each one of the seven ligands as a downloadable table. An interactive 3D viewer highlighting the selected pocket is also shown. Physicochemical properties for each ligand are also available via their respective “Details” button. All hits retrieved for the ligand transplantation option (B) are summarized in a table where users may select one entry at a time to display its ligands on the interactive 3D viewer. In both results pages, users may retrieve additional ADMET properties for each small molecule shown and customize their own molecular docking via AutoDock Vina (C).

There results for biological ligand classification summarizes the predicted probability scores for each of the seven ligands in a downloadable table (Figure 4A). A series of basic properties including Molecular Weight, number of Rotatable Bonds, LogP, and depiction of ligand using the SmilesDrawer JavaScript library,^14^ are available via the “Details” button. The input structure is shown in an interactive 3D viewer on the right side of the page with the selected pocket region highlighted.

For the Ligand Transplantation option (Figure 4B), all hits are summarized in table format including details on the PDB accession code, overall root mean squared error (RMSD) between input protein structure and the structure from which the transplanted ligand derives, and pocket RMSD which accounts for the degree of structural similarity between all backbone atoms with 6 Å from the transplanted ligand. Users may select entries from the table to have their respective ligands loaded to the input structure via the interactive 3D viewer. For each entry on the results table, users may predict pharmacokinetic properties via pkCSM.^15^ In addition, users can download their input structure with the transplanted ligands for a given entry from the table.

Molecule docking (pose optimisation) of transplanted ligands can be performed using Autodock Vina^16^ via the “optimize” button, except when the ligand is an ion such as calcium (CA), zinc (ZN), and manganese (MN). Here, users may customize the optimisation by selecting the number of poses, as well as the size of the box used as search space, exhaustiveness, and energy values to help guide pose optimization (Figure 4C). The center of the box, defining the search space for docking, is automatically set as the centroid of all atoms in the selected ligand. On the results page for ligand optimization, poses are summarized in table format showing their AutoDock vina affinity scores (kcal/mol). Users can also rapidly rescore selected poses using CSM‐Lig.^17^ Similarly, to the results for ligand transplantation, users can also visualize each pose and the input structure using the interactive viewer. All posts and input structure are available for download.

A comprehensive help page with information about all prediction types are available at https://biosig.lab.uq.edu.au/csm_potential, including videos with comprehensive information about submitting and analyzing results from CSM‐Potential.

## 3 CONCLUSION

Here we report CSM‐Potential2, a deep learning platform for the study of protein interactions from 3D structures. This work is an update to our previous deep learning approach for the identification of PPI binding sites and biological ligand classification, in which we included prediction of NA binding sites and ligand transplantation via sequence and structure similarity based on experimental structures from the PDB. Our platform aims to facilitate access by nonexpert users to powerful state‐of‐the‐art methods that will certainly enrich their study on how proteins interact with a variety of different molecules, consequently allowing us to better understand how these interactions occur. Our platform is freely available as an easy‐to‐use web server and API at https://biosig.lab.uq.edu.au/csm_potential.

## 4 MATERIALS AND METHODS

### 4.1 Predicting PPI binding sites and biological ligand classification

Prediction of PPI binding sites and biological ligand classification follows the protocol described in our previous implementation of CSM‐Potential. The dataset used to build our predictive model was derived from 4 databases: 8466 experimentally characterized structures retrieved from the PRISM database of nonredundant PPIs^18^; and 3536 transient interactions were taken from PDBBind,^19^ SabDab antibody: antigen database^20^ and the ZDock benchmark.^21^ Proteins were clustered at sequence level using CD‐HIT^22^ with a 30% sequence identity, resulting in 3362 unique proteins. Furthermore, hierarchical clustering using the AgglomerativeClustering method available in the Scikit‐learn Python library was then used to split the data into training (3004) and test (358) sets. Interface residues were defined based on the change in solvent‐accessible surface (SASA) upon complexation. This was done by comparing the difference in SASA, at the residue level, between individual unbound proteins and within the complex. As the number of non‐interface residues is generally much larger than the number of interface points, during training our neural network, a random number of non‐interface residues was selected until an equal number of positive and negative samples was achieved.

The dataset for biological ligand classification is derived from the PDB where at list one the following seven chemical compounds were present: Adenosine diphosphate, coenzyme A, Flavin adenine dinucleotide, heme (HEM), nicotinamide adenine dinucleotide, nicotinamide adenine dinucleotide phosphate or S‐adenosyl methionine (SAM). Despite being a small sample size representation of the full chemical space available to be explored, these seven compounds were selected due to their large structure availability. Proteins were then clustered at the sequence level using the PDB pre‐computed sequence clusters. The final dataset comprises 1468 structures, which were randomly split into training (72%) validation (8%), and testing (20%) sets.

For both predictive tasks, we trained end‐to‐end neural networks using the MaSIF framework,^11^ which decomposes proteins surfaces into overlapping patches based on a geodesic radius of 12 Å and then uses these in combination with geometric and chemical features to generate embeddings from learnable Gaussian Kernels.

### 4.2 Nuclei Acid binding site prediction

Prediction of NA binding sites is given based on the GraphBind^23^ implementation, an approach that uses a hierarchical graph neural network to learn local patterns for binding residue prediction. The dataset used to train GraphBind is derived from the BioLiP database^24^ and comprises 48 133 binding sites from 6342 protein‐NA complexes. (4344 DNA‐protein and 1558 RNA‐protein complexes). Entries were then divided into training and test sets based on their release date. Clustering at sequence level using CD‐HIT with a 30% similarity threshold and at structural level using TM scores >0.5 were performed in order to remove redundancy. In addition, data augmentation was applied as the number of NA‐binding residues is significantly smaller than the nonbinding ones. The final training set comprises 573 DNA‐binding and 495 RNA‐binding protein chains (20% of all entries in the training set were used as a validation set during the training of the network), while the test set comprised 129 DNA‐binding proteins and 117 RNA‐binding proteins.

After data curation, sequence, and structure‐based features were extracted, including pseudo‐positions, atomic features, secondary structure profiles, and evolutionary conservation generated using PSI‐BLAST and HHblits.^25^ Geometric knowledge is incorporated via a sliding sphere defined in the 3D space centered at each residue, including the physicochemical properties, which are then used to train a hierarchical graph neural network consisting of three modules: (i) a graph neural network encoder, used to generate the node feature vectors of the graph; (ii) a gated‐recurrent‐unit‐based graph neural network block, used to update the latent feature vectors of edges, nodes and the graph; and finally (iii) an MLP classifier used to make the predictions at a residue level.

GraphBind DNA binding site prediction performance was compared with seven other methods, outperforming all of them with a MCC (Matthews Coefficient Correlation) of 0.499 and AUC (Area Under the Curve) of 0.927. DNABind^26^ was the second‐best method, achieving MCC of 0.411 and AUC of 0.858. Performance assessment for RNA binding site prediction also shows GraphBind as the top performing method when compared with six other approaches, achieving MCC of 0.322 and AUC 0.854, followed by NucleicNet^27^ and aaRNA^28^ with MCCs of 0.788 and 0.771, respectively. Average performance metrics after 10 repetitions for all metrics have been compiled from the original study and summarized in Table 1. In addition, the original work discussed the performance of GraphBind on structures modeled using MODELLER,^29^ showing there was a drop in performance, which could be explained by the impact on the graphs built from the structural contexts, and consequently affecting the adjacency matrix from which the embeddings are derived.

**TABLE 1:** Performance comparison of GraphBind and other methods on the test sets for DNA and RNA binding site prediction.

	Method	Precision	Recall	F1	MCC	AUC
DNA	GraphBind	0.425	0.676	0.522	0.499	0.927
	TargetDNA	0.280	0.417	0.335	0.291	0.825
	TargetS	0.370	0.239	0.291	0.262	‐
	DNAPred	0.353	0.396	0.373	0.332	0.845
	SVMnuc	0.371	0.316	0.341	0.304	0.812
	COACH‐D	0.360	0.324	0.341	0.302	0.761
	NucBind	0.373	0.323	0.346	0.309	0.797
	DNABind	0.346	0.601	0.44	0.411	0.858
RNA	GraphBind	0.294	0.463	0.358	0.322	0.854
	RNABindRPlus	0.227	0.273	0.248	0.202	0.717
	SVMnuc	0.240	0.231	0.235	0.192	0.729
	COACH‐D	0.252	0.221	0.235	0.195	0.663
	NucBind	0.235	0.231	0.233	0.189	0.715
	aaRNA	0.166	0.484	0.247	0.214	0.771
	NucleicNet	0.201	0.371	0.261	0.216	0.788

Furthermore, given the unbalanced nature of the datasets used to train the neural network (most samples are residues not interacting with a nucleic acid), the authors demonstrate the impact of applying data augmentation on training both predictive models (DNA and RNA binding site identification). Results show an increase in Recall, F1 score, MCC, and AUC, indicating both models performed better in identifying true binding site residues. Average performance metrics after 10 repetitions for all metrics have been compiled from the original study and summarized in Table 2.

**TABLE 2:** Performance of GraphBind on the test set after before and after applying data augmentation on the training data.

	Method	Precision	Recall	F1	MCC	AUC
DNA	No augmentation	0.465	0.588	0.517	0.487	0.919
	Augmentation	0.425	0.676	0.522	0.499	0.927
RNA	No augmentation	0.306	0.404	0.346	0.307	0.849
	Augmentation	0.294	0.463	0.358	0.322	0.854

### 4.3 Ligand transplantation

Ligand transplantation is performed using a modification of the AlphaFill algorithm.^30^ Given an amino‐acid sequence, the program runs a BLAST search against all sequences available in the PDB‐REDO databank,^31^ and for each BLAST hit the algorithm checks for compounds of interest, which are then filtered via sequence identity using a cutoff of 25%. Each selected hit is then structurally aligned on the Cα of the residues that match the BLAST alignment. For each compound of interest in the hit list, the algorithm scans its local surroundings and all backbone atoms within 6 Å are used for a local structural alignment. Unless the same compound has already been transplanted within 3.5 Å of the centroid of the compound to be fitted or protein atoms are present within 4 Å from the atoms of the compound to be fitted, the compound is “transplanted” into the input structure. For converting files from PDB format to mmCIF, here we used the *pdb2cif* implementation from the cif‐tools suite of programs used to process protein structure data available at https://github.com/PDB-REDO/cif-tools.

The pose optimization feature in the server is implemented using AutoDock Vina (version 1.1.2)^16^ and OpenBabel (version 3.1.1) is used for converting protein and ligand structure files from PDB format to PDBQT format required by AutoDock Vina.

### 4.4 Webserver

The server backend is built using Flask version 1.12.5, a library from the Python programming language, while the front end is built using Materialize version 1.0.0. The server runs on a Linux machine running Nginx.

## AUTHOR CONTRIBUTIONS

**Carlos H. M. Rodrigues:** Methodology; software; validation; writing – original draft; formal analysis; investigation. **David B. Ascher:** Conceptualization; project administration; writing – review and editing; funding acquisition; formal analysis; supervision; investigation.

## CONFLICT OF INTEREST STATEMENT

The authors declare no conflict of interest.

## Data Availability

The data that support the findings of this study are openly available in CSM_Potential at https://biosig.lab.uq.edu.au/csm_potential.

## References

[1] Paumi CM , Menendez J , Arnoldo A , et al. Mapping protein–protein interactions for the yeast ABC transporter Ycf1p by integrated split‐ubiquitin membrane yeast two‐hybrid analysis. Mol Cell. 2007;26:15‐25. 17434123 10.1016/j.molcel.2007.03.011

[2] Nicod C , Banaei‐Esfahani A , Collins BC . Elucidation of host‐pathogen protein–protein interactions to uncover mechanisms of host cell rewiring. Curr Opin Microbiol. 2017;39:7‐15. 28806587 10.1016/j.mib.2017.07.005 PMC5732060

[3] Gao J , Li WX , Feng SQ , et al. A protein–protein interaction network of transcription factors acting during liver cell proliferation. Genomics. 2008;91:347‐355. 18255255 10.1016/j.ygeno.2007.12.007

[4] Chuderland D , Seger R . Protein–protein interactions in the regulation of the extracellular signal‐regulated kinase. Mol Biotechnol. 2005;29:57‐74. 15668520 10.1385/MB:29:1:57

[5] The UniProt Consortium . UniProt: the universal protein knowledgebase in 2021. Nucleic Acids Res. 2021;49:D480‐D489. 33237286 10.1093/nar/gkaa1100 PMC7778908

[6] Berman HM , Westbrook J , Feng Z , et al. The Protein Data Bank. Nucleic Acids Res. 2000;28:235‐242. 10592235 10.1093/nar/28.1.235 PMC102472

[7] Varadi M , Anyango S , Deshpande M , et al. AlphaFold protein structure database: massively expanding the structural coverage of protein‐sequence space with high‐accuracy models. Nucleic Acids Res. 2022;50:D439‐D444. 34791371 10.1093/nar/gkab1061 PMC8728224

[8] Lin Z , Akin H , Rao R , et al. Evolutionary‐scale prediction of atomic‐level protein structure with a language model. Science. 2023;379:1123‐1130. 36927031 10.1126/science.ade2574

[9] Murakami Y , Mizuguchi K . Applying the naive Bayes classifier with kernel density estimation to the prediction of protein–protein interaction sites. Bioinformatics. 2010;26:1841‐1848. 20529890 10.1093/bioinformatics/btq302

[10] Xue LC , Dobbs D , Honavar V . HomPPI: a class of sequence homology based protein–protein interface prediction methods. BMC Bioinformatics. 2011;12:244. 21682895 10.1186/1471-2105-12-244 PMC3213298

[11] Gainza P , Sverrisson F , Monti F , et al. Deciphering interaction fingerprints from protein molecular surfaces using geometric deep learning. Nat Methods. 2020;17:184‐192. 31819266 10.1038/s41592-019-0666-6

[12] Rodrigues CHM , Ascher DB . CSM‐potential: mapping protein interactions and biological ligands in 3D space using geometric deep learning. Nucleic Acids Res. 2022;50:W204‐W209. 35609999 10.1093/nar/gkac381 PMC9252741

[13] Rose AS , Bradley AR , Valasatava Y , Duarte JM , Prlic A , Rose PW . NGL viewer: web‐based molecular graphics for large complexes. Bioinformatics. 2018;34:3755‐3758. 29850778 10.1093/bioinformatics/bty419 PMC6198858

[14] Probst D , Reymond JL . SmilesDrawer: parsing and drawing SMILES‐encoded molecular structures using client‐side JavaScript. J Chem Inf Model. 2018;58(1):1‐7. 29257869 10.1021/acs.jcim.7b00425

[15] Pires DE , Blundell TL , Ascher DB . pkCSM: predicting small‐molecule pharmacokinetic and toxicity properties using graph‐based signatures. J Med Chem. 2015;58:4066‐4072. 25860834 10.1021/acs.jmedchem.5b00104 PMC4434528

[16] Trott O , Olson AJ . AutoDock Vina: improving the speed and accuracy of docking with a new scoring function, efficient optimization, and multithreading. J Comput Chem. 2010;31:455‐461. 19499576 10.1002/jcc.21334 PMC3041641

[17] Pires DE , Ascher DB . CSM‐lig: a web server for assessing and comparing protein‐small molecule affinities. Nucleic Acids Res. 2016;44:W557‐W561. 27151202 10.1093/nar/gkw390 PMC4987933

[18] Baspinar A , Cukuroglu E , Nussinov R , Keskin O , Gursoy A . PRISM: a web server and repository for prediction of protein–protein interactions and modeling their 3D complexes. Nucleic Acids Res. 2014;42:W285‐W289. 24829450 10.1093/nar/gku397 PMC4086120

[19] Wang R , Fang X , Lu Y , Yang CY , Wang S . The PDBbind database: methodologies and updates. J Med Chem. 2005;48:4111‐4119. 15943484 10.1021/jm048957q

[20] Raybould MIJ , Marks C , Lewis AP , et al. Thera‐SAbDab: the therapeutic structural antibody database. Nucleic Acids Res. 2020;48:D383‐D388. 31555805 10.1093/nar/gkz827 PMC6943036

[21] Vreven T , Moal IH , Vangone A , et al. Updates to the integrated protein–protein interaction benchmarks: docking benchmark version 5 and affinity benchmark version 2. J Mol Biol. 2015;427:3031‐3041. 26231283 10.1016/j.jmb.2015.07.016 PMC4677049

[22] Li W , Godzik A . Cd‐hit: a fast program for clustering and comparing large sets of protein or nucleotide sequences. Bioinformatics. 2006;22:1658‐1659. 16731699 10.1093/bioinformatics/btl158

[23] Xia Y , Xia CQ , Pan X , Shen HB . GraphBind: protein structural context embedded rules learned by hierarchical graph neural networks for recognizing nucleic‐acid‐binding residues. Nucleic Acids Res. 2021;49:e51. 33577689 10.1093/nar/gkab044 PMC8136796

[24] Yang J , Roy A , Zhang Y . BioLiP: a semi‐manually curated database for biologically relevant ligand‐protein interactions. Nucleic Acids Res. 2013;41:D1096‐D1103. 23087378 10.1093/nar/gks966 PMC3531193

[25] Remmert M , Biegert A , Hauser A , Soding J . HHblits: lightning‐fast iterative protein sequence searching by HMM–HMM alignment. Nat Methods. 2011;9:173‐175. 22198341 10.1038/nmeth.1818

[26] Liu R , Hu J . DNABind: a hybrid algorithm for structure‐based prediction of DNA‐binding residues by combining machine learning‐ and template‐based approaches. Proteins. 2013;81(11):1885‐1899. 23737141 10.1002/prot.24330

[27] Lam JH , Li Y , Zhu L , et al. A deep learning framework to predict binding preference of RNA constituents on protein surface. Nat Commun. 2019;10:4941. 31666519 10.1038/s41467-019-12920-0 PMC6821705

[28] Li S , Yamashita K , Amada KM , Standley DM . Quantifying sequence and structural features of protein‐RNA interactions. Nucleic Acids Res. 2014;42:10086‐10098. 25063293 10.1093/nar/gku681 PMC4150784

[29] Sali A , Blundell TL . Comparative protein modelling by satisfaction of spatial restraints. J Mol Biol. 1993;234:779‐815. 8254673 10.1006/jmbi.1993.1626

[30] Hekkelman ML , de Vries I , Joosten RP , Perrakis A . AlphaFill: enriching AlphaFold models with ligands and cofactors. Nat Methods. 2023;20:205‐213. 36424442 10.1038/s41592-022-01685-y PMC9911346

[31] van Beusekom B , Touw WG , Tatineni M , et al. Homology‐based hydrogen bond information improves crystallographic structures in the PDB. Protein Sci. 2018;27:798‐808. 29168245 10.1002/pro.3353 PMC5818736

